# Proposing DAPP-MR as a disaster risk management pathways framework for complex, dynamic multi-risk

**DOI:** 10.1016/j.isci.2022.105219

**Published:** 2022-09-30

**Authors:** Julius Schlumberger, Marjolijn Haasnoot, Jeroen Aerts, Marleen de Ruiter

**Affiliations:** 1 Deltares, Boussinesqweg 1, 2629 Delft, the Netherlands; 2 Institute for Environmental Studies (IVM), Vrije Universiteit Amsterdam, De Boelelaan 1111, 1081 Amsterdam, the Netherlands

**Keywords:** Earth sciences, Climatology, Environmental event

## Abstract

Climate change impacts are increasingly complex owing to compounding, interacting, and cascading risks across sectors. However, approaches to support Disaster Risk Management (DRM) addressing the underlying (uncertain) risk driver interactions are still lacking. We tailor the approach of Dynamic Adaptive Policy Pathways (DAPP) to DAPP-MR to design DRM pathways for complex, dynamic multi-risk in multi-sector systems. We review the recent multi-hazard and multi-sector research to identify relevant aspects of multi-risk management frameworks and illustrate the suitability of DAPP-MR using a stylized case. It is found that rearranging the analytical steps of DAPP by introducing three iteration stages can help to capture interactions, trade-offs, and synergies across hazards and sectors. We show that DAPP-MR may guide multi-sector processes to stepwise integrate knowledge toward multi-risk management. DAPP-MR can be seen as an analytical basis and first step toward an operational, integrative, and interactive framework for short-to long-term multi-risk DRM.

## Highlights

• Long-term DRM needs explicit attention to interactions across hazards and sectors
• Our pathways approach follows iterative stages to gradually integrate multi-risk
• Trade-offs and synergies across hazards and sectors influence sectoral DRM pathways

## Graphical abstract

**Figure:**
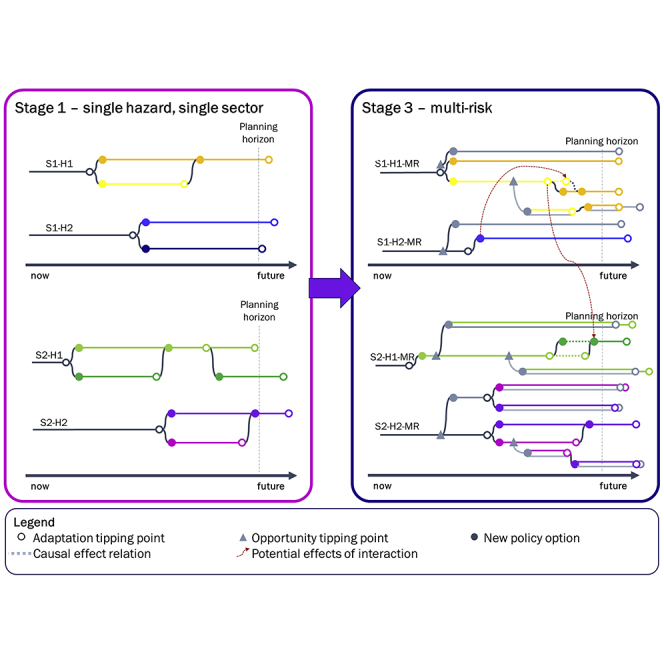

## Introduction

A variety of natural hazards continue to cause substantial damages. Since 1990, natural hazards have caused annual economic losses of about $260-310 billion globally (Ward et al., 2020). Between 1980 and 2015, economic damages by meteorological events alone totaled approximately 433 billion (in 2015 values) in the European Union (de Groeve et al., 2017). The risk of climate extremes will increase as climate change leads to further intensification of natural hazards (IPCC, 2021) while socioeconomic developments may change the exposure and vulnerability of populations, assets, and infrastructures toward a variety of natural hazards (Herman and Giuliani, 2018; Harrison et al., 2016).

In this context, the Intergovernmental Panel on Climate Change (IPCC) and the Sendai Framework in the context of UN Disaster Risk Reduction (UNDRR) have called for a multi-hazard and multi-sectoral perspective to reduce the impact of natural hazards and avoid maladaptation (UNDRR, 2017; IPCC, 2021). We follow the definition of *multi-hazard* as “the selection of multiple major hazards that the country faces, and the specific contexts where hazardous events may occur simultaneously, cascadingly or cumulatively over time, and taking into account the potential interrelated effects” (UNDRR, 2017). This definition is primarily used in contexts where multiple natural hazards play a role, including climate extremes and slow-onset events such as sea level rise (Gill and Malamud, 2014; Liu et al., 2016; van Westen and Greiving, 2017; Tilloy et al., 2019). Similarly, we define *multi-sector* in line with the concept of “system-of-systems” proposed by Maier (1998) as a set of socio-economic sectors, all possibly consisting of sub-systems characterized by stakeholders and other elements, which are interconnected beyond the sector boundaries, within and beyond the boundaries of the existing spatial system at stake (Reed et al., 2022; Klein and van Vliet, 2013). An example of a multi-sector system is described in the STAR Methods. Multi-hazard, multi-sector system characteristics can be captured by the term *complex multi-risk*, based on (Gallina et al., 2016, p. 125), where *multi-risk* “determines the whole risk from several hazards, taking into account possible hazards and vulnerability interactions entailing both a multi-hazard and multi-vulnerability perspective.” Complex multi-risk hereby explicitly acknowledges that the interactions in a multi-risk setting occur across sectoral boundaries. More key terms and definitions relevant in the context of multi-risk are summarized in Box 1.

Box 1 **Key terms and definitions in the context of multi-risk**

**Table:** 

Complexity: “A causal chain with many intervening variables and feedback loops that do not allow the understanding or prediction of the system’s behavior on the basis of each component’s behavior.” (Aven et al., 2018, p. 5)
Deep uncertainty: “That is, where analysts do not know, or the parties to a decision cannot agree on, (1) the appropriate conceptual models that describe the relationships among the key driving forces that will shape the long-term future, (2) the probability distributions used to represent uncertainty about key variables and parameters in the mathematical representations of these conceptual models, and/or (3) how to value the desirability of alternative outcomes.” (Lempert, 2003, p. 12)
Disaster Risk Management (DRM): “The application of disaster risk reduction policies and strategies to prevent new disaster risk, reduce existing disaster risk and manage residual risk, contributing to the strengthening of resilience and reduction of disaster losses.” (UNDRR, 2017)
Exposure: “The situation of people, infrastructure, housing, production capacities and other tangible human assets located in hazard-prone areas.” (UNDRR, 2017)
(Natural) Hazard: “A process, phenomenon or human activity that may cause loss of life, injury or other health impacts, property damage, social and economic disruption, or environmental degradation. Natural hazards are predominantly associated with natural processes and phenomena.” (UNDRR, 2017)
(Multi-)Hazard: “The selection of multiple major hazards that the country faces, and the specific contexts where hazardous events may occur simultaneously, cascadingly or cumulatively over time, and taking into account the potential interrelated effects.” (UNDRR, 2017)
Risk: “The potential loss of life, injury, or destroyed or damaged assets which could occur to a system, society, or a community in a specific period of time, determined probabilistically as a function of hazard, exposure, vulnerability and capacity.” (UNDRR, 2017)
System-of-system: “The term system of systems designates the case where the constituent elements are collaborating systems that exhibit the properties of operational independence (each constituent system operates to achieve a useful purpose independent of its’ participation in the system of systems) and managerial independence (each constituent system is managed and evolved, at least in part, to achieve its’ own goals rather than the system of systems goals).” (Maier, 1998, p. 271)
Vulnerability: “The conditions determined by physical, social, economic, and environmental factors or processes which increase the susceptibility of an individual, a community, assets, or systems to the impacts of hazards.” (UNDRR, 2017)
(Multi-)Vulnerability: “refers to (1) a variety of exposed sensitive targets (e.g. population, infrastructure, cultural heritage, etc.) with possible different vulnerability degree against the various hazards and (2) time-dependent vulnerabilities, in which the vulnerability of a specific class of exposed elements may change with time as consequence of different factors (e.g. the of other hazardous events).” (Gallina et al., 2016, p. 125)

The call for a multi-risk approach in DRM follows a growing body of literature that has identified limitations of the current single-hazard risk management approaches that lead to inappropriate risk reduction strategies or enhanced vulnerabilities within a system (Schipper, 2020; Ciurean et al., 2018; Duncan et al., 2016; de Ruiter et al., 2021; de Ruiter and van Loon, 2022; Suppasri et al., 2021; Koks et al., 2019; Goulart et al., 2021; Pourghasemi et al., 2020; Tabari et al., 2021). Recently, it has been proposed to account for the dynamics of multi-sector systems and responses to risk improving our current static understanding of risk to define successful long-term risk management strategies (Simpson et al., 2021, 2022, *in review*; Reisinger et al., 2020). The success of such strategies is also influenced and valued by various stakeholders because of their potentially contested objectives and intensifying conflicts about limited resources (Reed et al., 2022; Palutikof et al., 2019).

While multi-risk assessment has been advanced in recent years, a systematic approach to support action-oriented DRM decision-making accounting for complexity, ambiguity, and uncertainty is still missing (Schinko et al., 2017; Ward et al., 2022; Aven and Renn, 2020). However, support for short-to long-term risk reduction is needed as the dynamic feedback between hazards and responses of sectors makes DRM decision-making complex and deeply uncertain (UNDRR, 2017; Magnan et al., 2020). Such deep uncertainties (Lempert, 2003) are for example caused by the lack of consensus about certain cause-–effect relations in the system, or by the non-stationary and chaotic dynamics of future changes in the climate and socio-economic systems (Haasnoot et al., 2013; Kwakkel et al., 2015; Herman and Giuliani, 2018; Baldwin, 2021; Wright et al., 2019; Molenveld et al., 2020; Bosomworth et al., 2017).

Dynamic Adaptive Policy Pathways (DAPP) is a framework that addresses dynamic systems and helps to design long-term strategies that can be broken into manageable steps to be implemented and adapted over time. DAPP produces and evaluates alternative sequences of policy actions (called ’pathways’, see Box 2 for key definitions) under a range of (transient) scenarios. Pathways typically start with low-regret actions that are robust and/or flexible to further adapt. Transient scenarios describe the variety of plausible temporal developments of hazards, exposure, and vulnerability over the considered time horizon driven by climate change and socio-economic developments (Haasnoot et al., 2013). System parameters are used as signposts to identify when a system is approaching an Adaptation Tipping Point (ATP) which requires the implementation of additional policies to further comply with pre-defined system (performance) objectives. As such, DAPP supports decision-making under deep uncertainty by designing strategies that perform well in a wide range of plausible futures, and that can be adapted based on monitoring the changes and advanced knowledge in the real-world system (Haasnoot et al., 2013; Lawrence et al., 2018; Kwakkel et al., 2015). DAPP can follow a phased approach and uses various sources of evidence with increasing levels of detail (from narratives to sophisticated computer models). As such, DAPP is able to deal with limited knowledge of varying sources including different requirements for data, time, and other resources (Haasnoot et al., 2019; Werners et al., 2021b).

Box 2 **Key terms and definitions in the context of DAPP**

**Table:** 

Path dependency: Path dependencies can be caused by a) certain scenarios with a critical event (like an extreme flood or drought event) or because of b) self-reinforcing mechanisms (e.g. rigid institutionalization of risk governance) and effect of previous decisions (e.g., large investment in a storm surge barrier) (Sydow et al., 2009; Hanger-Kopp et al., 2022).
(Adaptation) Pathway:“A series of adaptation choices involving trade-offs between short-term and long-term goals and values. These are processes of deliberation to identify solutions that are meaningful to people in the context of their daily lives and to avoid potential maladaptation.” (IPCC, 2022, p. 2917)
Policy: “Policy is the development, enactment, and implementation of a plan or course of action carried out through a law, rule, code, or other mechanism in the public or private sector.” (Bogenschneider and Corbett, 2010, p. 3)
(Adaptation) Tipping Point: “An adaptation tipping point (ATP) is the moment when the magnitude of change is such that a current management strategy can no longer meet its objectives. As a result, adaptive management is needed to prevent or postpone these ATPs.” (Nanda et al., (2018), p. 3), based on Kwadijk et al., (2010))
(Opportunity) Tipping Point: “Points at which a particular action becomes feasible or attractive, for example because of lower costs of actions or technical developments.” (Haasnoot et al., 2019, p. 86)
Transient scenario: “Represent a variety of relevant uncertainties and their development over time.” (Haasnoot et al., 2013, p. 489)

**Figure 1:**
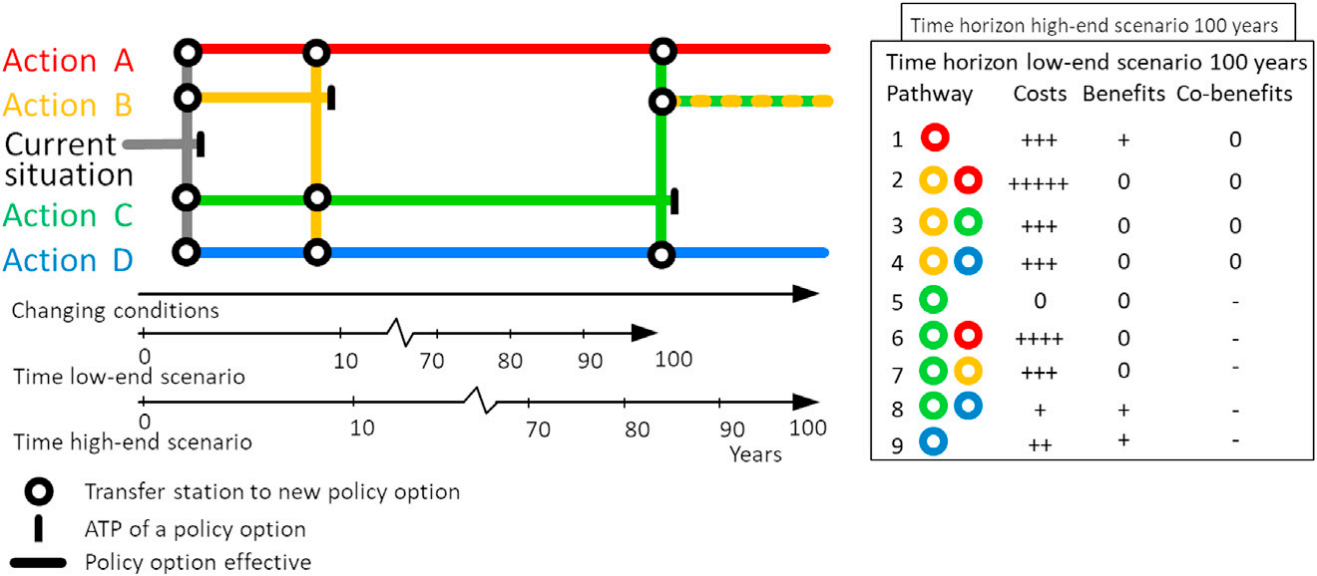
Example adaptation pathways map along with a scorecard for qualitative analysis of costs and benefits of chosen pathways The pathways map indicates several possible routes to get to a desired point (target) in the future. The timing of decision points is dependent on the transient scenario indicated by two timelines with different timescales. Circles indicate unidirectional transfer stations and lines represent routes through time. Blocks indicate terminal stations at which an ATP is reached and new policies need to be implemented. Map modified from Haasnoot et al. (2013).

DAPP methods have proven useful to make decision options and their effects comprehensible and accessible (Lawrence and Haasnoot, 2017). One of the key strengths of DAPP is the explicit consideration and visualization of decision-making over time in the form of metro-like maps of pathways as shown in Figure 1. The pathways map is the main product to support policy analysis by visualizing different strategy options, allowing laypeople to unravel the complexity of the analysis. For example, by considering various transient scenarios, DAPP provides plausible implementation time frames for specific policy options (instead of one definite point) as part of a specific policy pathway (Lawrence and Haasnoot, 2017). Thus, DAPP helps identify path dependencies, which reduce future decision options and ultimately lead to suboptimal, persistent outcomes, also called ’lock-ins’ (Hanger-Kopp et al., 2022; Arthur, 1989). The pathways map is often accompanied by a scorecard used to comprehensively present benefits, costs, and other decisive criteria for the various plausible pathways.

DAPP has been applied in a wide field of single-hazard considerations using a variety of qualitative and quantitative stakeholder-driven co-production processes: river flood management (Buurman and Babovic, 2016; Haasnoot et al., 2013; Ranger et al., 2013; Jordhus-Lier et al., 2019), drought management (Haasnoot et al., 2014; Vizinho et al., 2021; Totin et al., 2021; Burnham and Ma, 2018), coastal planning (Haasnoot et al., 2019; Kwakkel et al., 2016; Baldwin, 2021; Abel et al., 2016; Lawrence et al., 2018; Ramm et al., 2018a, 2018b; Barnett et al., 2014; Buijs et al., 2018; Kuipers, 2017; Townend et al., 2021) and other applications (Costa et al., 2021; Jacobs et al., 2019; Skrimizea and Parra, 2020; Jafino et al., 2021; Katz, 2020). In its applications, the limitations of the framework have been widely discussed: current applications of the framework simplify complex problems and thus neglect relevant dynamic interrelations (Bosomworth et al., 2017; Jafino et al., 2019) and contested objectives (Kwakkel et al., 2016; Head, 2019; Abel et al., 2016) within multi-risk contexts, or may face challenges in reporting their findings in a transparent and comprehensive way (Shavazipour et al., 2021). As such, DAPP is not yet capable of dealing with the complexities and interdependencies of multi-hazard, multi-sector systems.

The main objective of this article is to tailor the analytical framework of DAPP to design DRM pathways in the context of complex and dynamic multi-risk settings. This tailored version is called DAPP-MR (DAPP for multi-risk). Relevant aspects to characterize multi-risk systems presents relevant aspects for characterizing multi-hazard, multi-sector systems. Integrating multi-risk elements into the DAPP framework presents the analytical framework of DAPP and how complex multi-risk elements can be incorporated into this framework. The DAPP-MR framework presents and discusses the proposed DAPP-MR framework. In Testing the framework in a stylized case a stylized case is used to test the utility of some elements of DAPP-MR. Conclusions and limitations of this study discusses the key findings, limitations, and open research questions.

## Relevant aspects to characterize multi-risk systems

In recent literature, three themes are detectable that are relevant to characterize multi-hazard and multi-sector interactions to design pathways: 1) Effects of multiple, interacting hazards, 2) Dynamics and interdependencies of sectors, 3) Trade-offs and synergies of DRM policy options across different sectors and different spatial and temporal scales. In the following section, we provide more detailed elaborations on relevant aspects of each of the themes.

### Effects of multiple interacting hazards

To account for the potential effects of interacting hazards while developing adaptation pathways, it is important to be able to characterize the natural hazards (see Box 3 for key definitions) in terms of hazard drivers, and hazard-related impact drivers (Murray et al., 2021) and their temporal and spatial scales to identify where interactions of hazards-related impact drivers can be expected (Gill and Malamud, 2014; de Angeli et al., 2022). This information is necessary to understand what hazard processes influence the extent and severity of certain hazards (e.g. floods: flow velocity, inundation depth, and duration). We define hazard-related impact drivers as a property of the hazard that can be directly linked to a certain (adverse) effect on the physical properties of an exposed element. Additionally, the level of correlation between hazards is important: uncorrelated hazards will lead to random interactions, while bidirectionally correlated hazards (e.g. owing to the same or correlated drivers) or uni-directionally correlated hazards (e.g. one hazard might trigger another one) influence the probability of the combined occurrence (Ciurean et al., 2018; Gill et al., 2020; Tilloy et al., 2019; Bevacqua et al., 2021). Nevertheless, the characterization of hazard interdependencies and quantification of hazard interaction effects are still a developing research field (Zscheischler et al., 2020; Tilloy et al., 2019; Ward et al., 2022). Consequently, using or adapting existing knowledge on hazard interactions also requires careful consideration of the related uncertainties. As a result of the hazard interactions, two types of impact interactions can be identified in literature which have been visualized in various ways (see e.g. Figure 2 in Balch et al. (2020), Figures 2–13 in Rimmer et al. (2022) or Figure 5 in Matanó et al. (2022):

1. interacting impacts co-occurring in space and time can be aggravating and result in larger disasters (Simpson et al., 2021; Reichstein et al., 2021) depending on the spatiotemporal variable exposure and vulnerability of exposed elements (Gallina et al., 2016; Liu et al., 2015; Simpson et al., 2021).
2. consecutive impacts resulting from a sequence of hazard events can have aggravating effects on a given exposed element depending on the temporal scales of the impacts, recovery, and persisting effects on the exposed element (de Ruiter et al., 2020; de Angeli et al., 2022).

Box 3 **Additional terms and definitions relevant in the context of multi-hazard effects and responses**

**Table:** 

(Disaster) Impact: “The total effect, including negative effects (e.g., economic losses) and positive effects (e.g., economic gains), of a hazardous event or a disaster. The term includes economic, human and environmental impacts.” (UNDRR, 2017)
(Disaster) Recovery: “The restoring or improving of livelihoods and health, as well as economic, physical, social, cultural and environmental assets, systems and activities, of a disaster-affected community or society, aligning with the principles of sustainable development and ’build back better’, to avoid or reduce future disaster risk.” (UNDRR, 2017)

### Dynamics and interdependencies of sectors

In DRM literature, the interrelations of stakeholders are mostly linked to impact interrelations, driven by connectedness and multi-vulnerability characteristics (Gallina et al., 2016; Simpson et al., 2021). So-called cascading impacts can have non-linear indirect effects on the system by causing additional damages and escalation points (Pescaroli and Alexander, 2018; Carter et al., 2021; Terzi et al., 2019). Such cascading impacts are not necessarily limited to one location, particularly if critical infrastructure is involved (Verschuur et al., 2022). Furthermore, the time -delays of cascading impacts might contribute to impact interactions as presented in the previous section (Pescaroli and Alexander, 2018; Amon et al., 2017). Those additional impacts are mostly driven by the existing interrelations within the system.

Different types of multi-sectoral interrelations make sectoral systems differently prone to impact-driven interrelations. Reed et al. (2022) highlight the importance of considering decision-making beyond risk management, as it drives changes in existing systems. It, therefore, affects the exposure and vulnerability of interdependent system elements, influencing not only the individual risk of a sector but also the cross-sectoral systemic risk (Hochrainer-Stigler et al., 2020) (see Box 4 for key terms). In the literature, four different intersectoral interrelations are discussed (Rimmer et al., 2022):

1. One system is connected to another system and relies on it to operate (Rinaldi et al., 2001; Galbusera et al., 2020).
2. Spatial proximity can lead to bi-directionally correlated responses in multiple systems (Dawson, 2015; Crona et al., 2021).
3. Shared markets, implications of post-disaster consumption behavior (Lawrence et al., 2020), and business interruptions can have consequences across scales (Meyer et al., 2013; Verschuur et al., 2022; Sillmann et al., 2022).
4. Governance structures including, local communities (Gupta and Sharma, 2006), play a role in vulnerability characteristics and the effectiveness of response and recovery efforts (Dawson, 2015; Rana et al., 2020; Ali et al., 2021).

Box 4 **Key terms and definitions used in the context of multi-sector systems**

**Table:** 

Power: The (in)capacity of actors to mobilize means to achieve ends (Avelino, 2021).
Synergy: The pursuit of some targets can create additional resources that facilitate the pursuit of other targets. When attempting to achieve one target improves other targets, that target has high pursuit synergies. If achievement of one goal becomes easier as other goals are approached or achieved, that goal is synergy (Moyer and Bohl, 2019).
Systemic risk: “Risk that is endogenous to, or embedded in, a system that is not itself considered to be a risk and is therefore not generally tracked or managed, but which is understood through systems analysis to have a latent or cumulative risk potential to negatively impact overall system performance when some characteristics of the system change.” (UNDRR, 2019, p. 45).
Trade-off: We define trade-offs in two ways. Interventions aimed at achieving one target that undermine the ability to pursue a different target have high pursuit trade-offs. Second, an target can be harder to achieve when other goals are pursued. These targets are trade-off vulnerable (Moyer and Bohl, 2019).

Conversely, integrating multiple sectors and their interrelations in risk governance also provides the opportunity to coordinate DRM across multiple stakeholders and sectors. As risk management is often seen as a community issue, the cooperation of various stakeholders has been highlighted to overcome widely acknowledged barriers such as limited resources, knowledge, or mandates (Butler et al., 2015; Scolobig et al., 2017). Cross-sectoral coordination could provide leverage to joint investments (Gold et al., 2022), offer flexibility and a wider range of adaptation options, enhance capacities to divert exposed assets to other management options, and implement policy option compromises with small trade-offs across sectors (Reed et al., 2022). However, identifying potential for cross-sectoral coordination entails additional challenges: trade-offs and synergies of DRM policy options may be different across sectors and thus may pose difficulties to the cooperative strategy of potentially competing interests (Aven and Renn, 2018; Gold et al., 2022). Additionally, the perception of risk might vary across sectors in spatiotemporal dimensions and thus influence the willingness to coordinate to take DRM actions (Di Baldassarre et al., 2021). Furthermore, accounting for the long-term performance of certain measures could lead to suboptimal short-term outcomes for stakeholders, highlighting the challenge to balance stakeholder needs across different temporal scales (Butler et al., 2020; AghaKouchak et al., 2020). Moreover, power dynamics will inherently affect the collaboration process, the development of cooperative strategies, and the cooperative stability of compromises (Hilhorst et al., 2020; Colloff et al., 2021; Aven and Renn, 2018). Thus, power dynamics shape the distributive justice and fairness of the developed pathways, such as the inclusion and valuation of perspectives of certain stakeholder groups or the distribution of impacts and benefits (Thomas et al., 2019; Araos et al., 2021; Head, 2019; Orlove et al., 2020).

Traditional approaches have assumed the stability of complex systems which allowed focusing on certain aspects of sub-systems (e.g. flood risk to residential areas). However, this assumption has been challenged because of unstable dynamics and observed non-linear effects in our increasingly interconnected world (Hochrainer-Stigler et al., 2020). Particularly when analyzing existing systems for longer time periods, the assumption of stable systems does not hold: new stakeholders appear, connections within and between sectors change following socio-economic, technological, and political developments within their system or broader context (Reed et al., 2022). Additionally, interdependencies within an existing system are not only affected by long-term developments, but also by short-term dynamics of e.g. vulnerability characteristics (Menk et al., 2022; de Ruiter and van Loon, 2022), effects of institutionalized governance structures and rebound effects in processes of decision making (Head, 2019; Siders and Pierce, 2021). In combination with a diverse understanding of interlinkages and interdependencies within systems (Menk et al., 2022; Hochrainer-Stigler et al., 2020) above mentioned challenges imply that accounting for dynamics and interdependencies of sectors, also requires diligent consideration of additional sources of (deep) uncertainty.

### Trade-offs and synergies of Disaster Risk Managementpolicy options

As mentioned in the previous section, the choice of policy options can influence the collaborative stability of the cross-sectoral process. Adaptation or risk reduction measures cannot be considered in isolation because of synergies across time, space, and sectors (Magnan et al., 2020; Gold et al., 2022; Schipper, 2020) as well as because of potential asynergies, defined as “the potential adverse effects of measures aimed to decrease the risk of one hazard on the risk of another hazard” (de Ruiter et al., 2021, p. 1). These trade-offs and synergies require the balancing of needs and interests beyond risk management (Buurman and Babovic, 2016). Different stakeholders will have mandates and resources for different policy options and might value the benefit of adaptation differently in different contexts (Lorenz et al., 2019; Magnan et al., 2020). Similarly, certain policy options could decrease in effectiveness over time, become unavoidable in the future, be not readily available now or become stranded assets (Magnan et al., 2020). Consequently, policy options should be characterized in terms of their potential effectiveness, readiness, lead time until full effectiveness, duration of benefits, societal acceptability, governability, potential co-benefits, and potential negative collateral effects (Magnan et al., 2020). In this way, strategies can be designed that pose no regret, are reversible/soft, have safety margins, reduce the decision-making time horizons, or are considerate of conflicts and synergies between strategies (Hallegatte and Przyluski, 2010).

However, assessing adaptation options is the first step in a process where different adaptation options are evaluated against each other. For such, often complex evaluation processes, a good understanding of the decision context and multi-vulnerabilities is necessary to avoid maladaptation, identify path dependencies and identify hard and soft adaptation limits (Schipper, 2020; Simpson et al., 2022, *in review*). Yet, the effects of risk management measures are still poorly understood, and lack of tools to quantify their effects on vulnerability as a systematic global stocktake on adaptation measures suggests (Berrang-Ford et al., 2021; Lorenz et al., 2019). In that context, Simpson et al. (2021) highlight that the financial, political, reputational, and technological risk of adaptation measures have received significant attention in recent research and public discussions. The lack of knowledge about the effectiveness of adaptation measures in terms of their temporal, spatial, and stakeholder-focused effects limits confidence in policy options (Magnan et al., 2020). As a result, not only the effectiveness and vulernability of policy options but also the perception and preferences are subject to (deep) uncertainties.

## Integrating multi-risk elements into the DAPP framework

**Figure 2:**
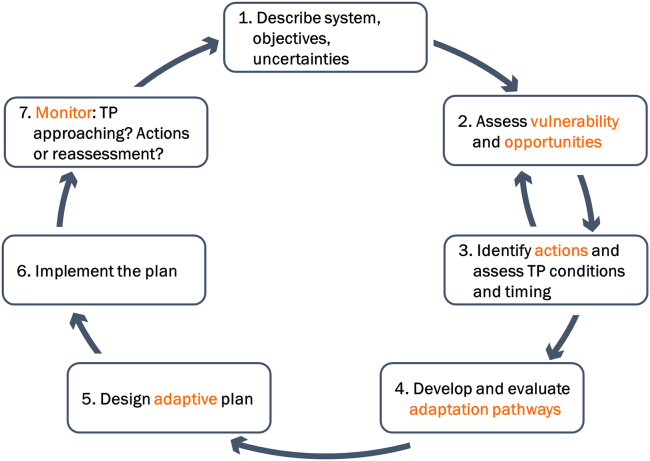
Analytical framework of DAPP Adapted from Haasnoot et al. (2019).

In the previous section, we discussed three themes and various relevant aspects that need to be addressed in DAPP-MR. In this section, we analyze how these aspects can be integrated into the DAPP framework. DAPP is an analytical framework consisting of seven steps, as shown in Figure 2. In the first step, the system and its decision context are characterized using methods for participatory problem framing. This step identifies not only the objectives and constraints in the system but also the set of transient scenarios (see Box 5 for key terms related to DAPP) which capture the range of plausible future evolution of the system. In the second step, vulnerabilities and opportunities in the system are identified to determine parameters and threshold values as adaptation tipping points (ATPs), which indicate the need for additional measures, and opportunity tipping points (OTPs), which indicate possibilities to leverage certain changes in the system for additional actions. Using transient scenarios, the relative timing of reaching these tipping points is determined, to inform the process of identifying contingent actions in Step 3. In Step 4, pathways are designed using various methods (from storylines to exploratory modeling) where ATPs and OTPs determine alternative routes depending on available and useful actions. Those pathways are then evaluated according to the initially defined main objectives as well as costs, co-benefits, and trade-offs. Furthermore, pathways are evaluated upon their feasibility to be implemented, the flexibility to further adapt and shift to other pathways if needed, and the robustness to perform well under a range of plausible future states. Whether follow-up adaptation options, when approaching an ATP, are still possible also depends on the time available to plan and implement them, which then determines when decisions need to be taken and in some case may mean a temporarily reduced performance of pathways. The feasibility of implementation can (sometimes) be shaped through supporting actions changing political support, technical innovation, and law and regulations (Haasnoot et al., 2020). In Step 5, adaptive strategies are designed, meaning that initiating decision points are determined based on preferred initial actions, long-term options, and potential signposts to ensure the flexibility of the identified strategy. Furthermore, contingency actions are considered to ensure the feasibility of potential future actions. A monitoring plan is also set up. In Steps 6 and 7, the strategy is implemented and monitored. The steps are described in detail by Haasnoot et al. (2019).

Box 5 **Key terms and definitions in the context of DAPP**

**Table:** 

Flexibility: “The inherent ability of the human and physical elements of a system to cope with, or adapt to, uncertain and changing conditions, in a timely and cost-effective manner.” (DiFrancesco and Tullos, 2014, p. 1528).
Robustness: “The insensitivity [of a system] to future conditions and the ability to perform satisfactorily over a broad range of future conditions.” (Beh et al., 2015, p. 1534).
Scenario: A plausible description of how the future may develop based on a coherent and internally consistent set of assumptions about key driving forces (e.g., rate of technological change (TC), prices) and relationships. Scenarios are neither predictions nor forecasts but are used to provide a view of the implications of developments and actions (IPCC, 2021).

### Analytical steps for DAPP-MR

In this section, we assess the capability of the analytical steps of DAPP to integrate the three themes. Table 1 summarizes the identified relevant aspects of multi-risk systems, grouped according to the three themes discussed in Relevant aspects to characterize multi-risk systems. These aspects were attributed to one or more analytical steps of DAPP. This was done by analyzing whether a certain aspect contributes information directly relevant to the analytical step (indicated with an “X” in Table 1). In this way, aspects insufficiently captured by the DAPP steps are identified.

**Table 1:** Summary of relevant aspects of multi-risk systems and their attribution to the analytical steps

		Key elements	1	2	3	4	5	6	7
Multi-hazard dynamics	∗	Hazard drivers	x		x				
		Temporal and spatial scales of hazard(s)	x	x					
	∗	Hazard-related impact drivers (spatial variable)		x	x				
		Type of hazard interaction	x						
		Level of hazard correlation	x	x	x				
	∗	Impact drivers of natural hazard(s)		x					
		Effect of hazard interaction on hazard-related impact drivers		x					
		Spatial variability of hazard-related impact driver interaction		x	x	x		x	
	∗	Vulnerability of element(s) to multi-hazard-related impact driver(s)		x					
		Dynamics of exposure, vulnerability & multi-hazard related impact drivers		x	x	x		x	
	∗	(Deep) uncertainties regarding hazard characteristics	x	x					
		(Deep) uncertainties regarding hazard interaction (effects)	x	x					
Interrelations of sectors	∗	Sector-specific objectives & perceptions (multi-temporal dimensions)	x		x	x	x	x	x
		Interdependencies between stakeholder objectives	x				x		
		Power dynamics between stakeholders	x				x	x	x
		Type of cross-sectoral interdependencies		x	x				
		Dynamics of exposure and vulnerability across exposed elements		x		x		x	
		Potential (dynamics of) spill-over chains		x	x				
		(Deep) uncertainties (as drivers of changes) in interrelations across sectors	x	x	x				
DRM policy options	∗	Stakeholder mandates for PO			x		x	x	x
	∗	Stakeholder preferences for PO				x	x		x
	∗	Characteristics & effects of PO			x	x	x		
		Vulnerability of PO regarding multi-hazard impact drivers			x				
		Trade-offs & synergies of PO across sectors		x	x		x		
		Trade-offs & synergies of PO across hazards		x	x		x		
		Collaborative potential regarding PO				x	x		
		(Deep) uncertainties of PO effectiveness & vulnerability			x				
		(Deep) uncertainties regarding PO perception & preferences			x	x	x		

Aspects marked with ∗ are already accounted for in DAPP. Numbered columns represent different analytical steps of the DAPP framework as proposed by Haasnoot et al. (2019). 1: Define decision context, 2: Assess vulnerabilities & opportunities, 3: Identify & evaluate policy options (POs), 4: Develop & evaluate adaptation pathways, 5: Design adaptive plan, 6: Implement the plan, 7: Monitoring.

Some of the multi-risk aspects in Table 1 are marked with an asterisk to indicate that they are not specific to multi-risk systems but are partly relevant in the original DAPP as well. Previous applications of DAPP incorporated knowledge about the vulnerability, exposure, and impact of one hazard at a time, identified the relevant decision context in order to limit potential policy choices, then added additional criteria for the selection and evaluation of a set of preferred pathways. For multi-risk considerations, the effort not only adds up linearly depending on the number of hazards and sectors but requires additional considerations to incorporate the increase in hazard- and vulnerability-related interactions and in the required performance evaluation of all policy options. Consequently, the amount of information gathered per analytical step increases significantly as shown in Table 1. Additionally, the number of required iterations across analytical steps is expected to increase, particularly related to multi-vulnerability characteristics and multi-sector pathway evaluations. For example, updating information between vulnerability and potential policy options requires more iterations when accounting for many more interdependent elements that are at risk with varying vulnerability toward different multi-hazard related impact drivers. Moreover, the level of conflict between the objectives of different sectors might influence the number and means of iterations required to reach a compromise and update the initially identified set of policy options.

### Implications of interdependencies for the timing of adaptation tipping points and opportunity tipping points

**Figure 3:**
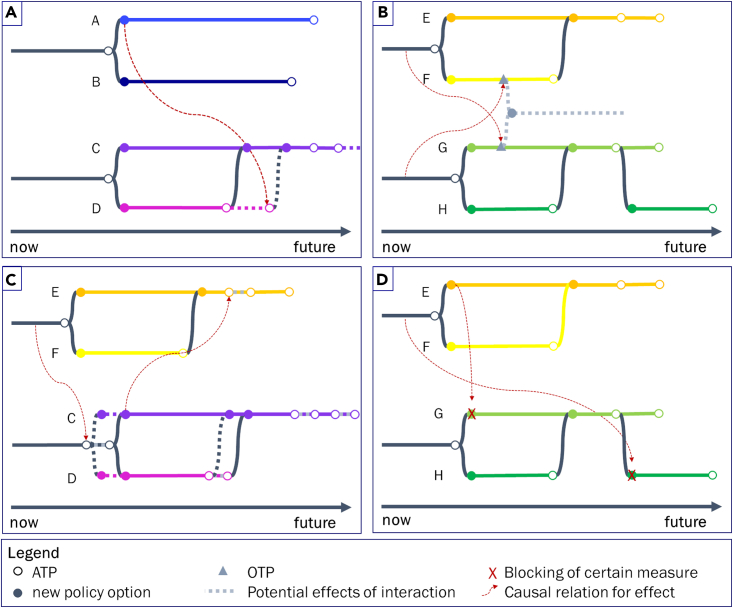
Set of illustrative combinations of two pathways The colored lines represent alternative portfolios of policy options (options A, B, C, D, E, F, G, and H). The panels visualize the potential interaction effects on the timing of ATPs and OTPs caused by hazard interactions, cross-sectoral interdependencies, and policy option interactions: (a) delaying ATPs, (b) new OTPs, (c) rushing ATPs, (d) and blocking of ATPs.

Many of the identified aspects of multi-risk systems touch upon the spatial and temporal dynamics of vulnerabilities and opportunities, which determine the ATPs and OTPs. Therefore, we investigated whether ATPs and OTPs are capable of dealing with the increased level of interdependence. In Figure 3, four different pathways are grouped together in varying combinations illustrating the implications of hazard interactions, cross-sectoral interdependencies, and policy option interactions on the shape of different pathways. Colors represent different policy options. New policy options are implemented after an ATP (circle) or OTP (triangle) is reached. Dotted lines indicate the potential effects of interactions (red dashed lines) on pathways, ATPs and OTPs. Generally, four effects can be identified:

• The **timing of ATPs can be delayed** (circle moves to the right) because of synergies between policy options (Figure 3A).
• **New OTPs can emerge** for various reasons (Figure 3B), e.g. multi-sector synergies could lead to additional available resources or willingness to cooperate in other ways to implement policy options that would not be feasible otherwise. Also, multi-hazard synergies (e.g., increased risk awareness) could reduce resistance regarding certain protection measures.
• Conversely, the **timing of ATPs can occur earlier** (circle moves to the left) when trade-offs between different policy options lead to asynergies or effects of multi-hazard interactions that exacerbate impacts (Figure 3C).
• Finally, certain **policy options can be inhibited** (red cross cuts off pathway) because of trade-offs, meaning that only one of the two measures can be implemented because of political, financial, or spatial reasons Figure 3D). Policy options can also be inhibited by multi-sector trade-offs resulting from contradicting objectives or perspectives.

The main implication of hazard interactions, cross-sectoral interdependencies, and policy option interactions is that ATPs and OTPs can capture the implications of enhanced interactions in multi-risk systems. We show that placing two pathways next to each other can support identifying trade-offs and synergies of certain policy options across hazards and sectors. For example, policy option A (light blue) in Figure 3A has a positive effect on the performance of policy option D (pink; enhanced lifetime before ATP is reached). Consequently, option A might be preferred over option B by the decision maker given the synergy across policy options (in addition to the slightly longer lifetime of option A). However, such a pairwise analysis of interactions between pathways might be sufficient as long as only a few individual pathway maps need to be considered. In more complex systems with multiple sectors and multiple pathways for each hazard, a decision-maker could easily lose track of which interactions exist and which cause more significant trade-offs or synergies.

## The DAPP-MR framework

**Figure 4:**
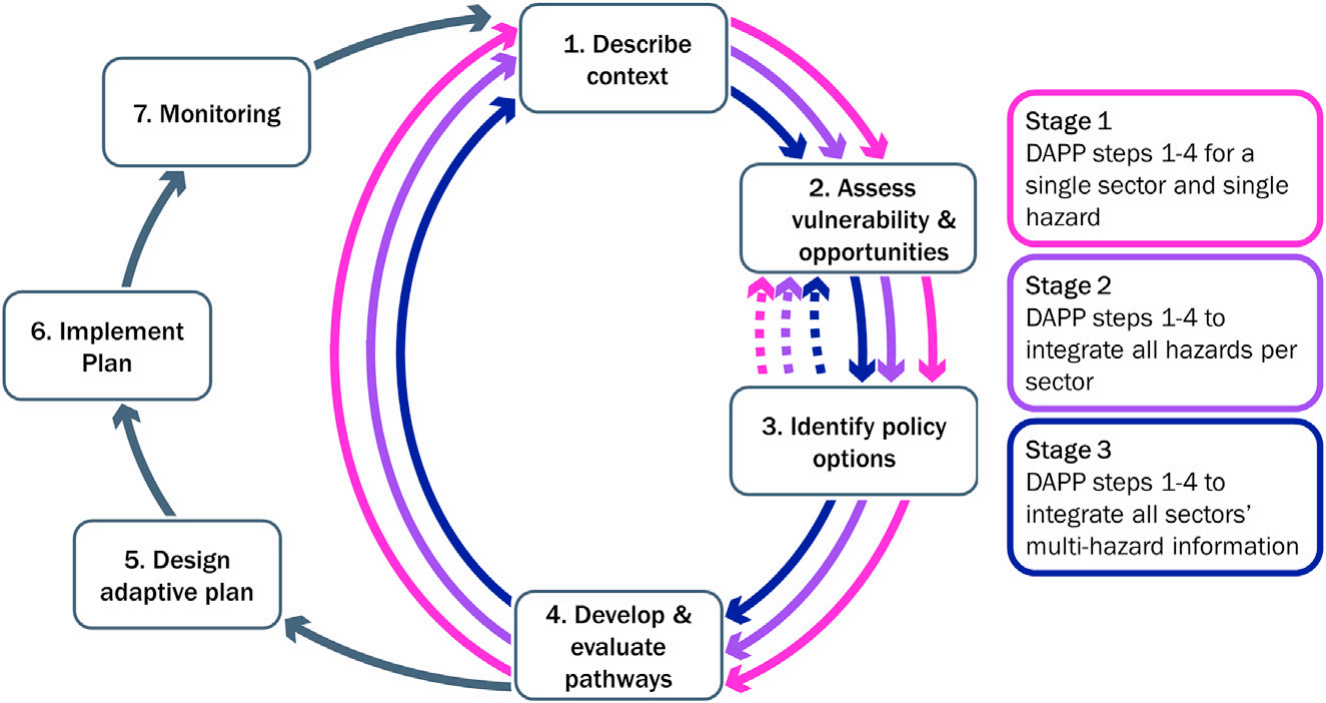
The proposed DAPP-MR framework It uses a rearranged set of analytical steps of the DAPP framework: Steps 1-4 are addressed in an interactive, staged approach to guide the integration of knowledge from a single-sector, single-hazard perspective (pink arrows), to a single-sector, multi-hazard perspective (purple arrows), to a multi-sector, multi-risk perspective (dark blue arrows) before advancing to Steps 5-7. Adapted from (Haasnoot et al., 2013, 2019).

In the previous sections, we showed how to enrich DAPP with contextual multi-risk elements without changing its step-wise approach. Furthermore, we discussed that the increased amount of information and cross-step interconnectedness may require additional, iterative considerations when developing DRM pathways for complex, dynamic multi-risk. Accordingly, we propose DAPP-MR consisting of a rearrangement of the seven steps of DAPP, as shown in Figure 4. In addition to the original iterative steps of DAPP, three stages of iterations are included to characterize the decision context, vulnerabilities, and opportunities, potential promising policy options and promising pathways:

• Stage 1: DAPP-MR starts with a single-sector, single-hazard perspective.
• Stage 2: Subsequently, all single-hazard considerations are integrated per sector to result in a single-sector, multi-hazard perspective.
• Stage 3: The single-sector, multi-hazard information is integrated into a multi-sector, multi-hazard perspective.

The additional three stages aim to support users with the integration of information from various knowledge sources to characterize the complex and interactive system before an adaptive plan of a certain strategy (Step 5, including preferred first actions, adaptive subsequent choices, and a plan to update the strategy depending on monitoring of the real-world system) can be developed. Although alternative ways to integrate the additional required information were considered, the proposed staged approach was identified as the most suitable one. For example, an alternative approach to integration could be step-wise by integrating single hazard, single sector, and multiple-risk information within each step. However, it was found to be much more difficult to keep track of all potential interactions when progressing to the next analytical step. The objective of the analysis was less clear. In combination with the absence of broad awareness for multi-risk across sectors as identified, this approach of integration is less favorable. The proposed framework proposes a sector-focused stage 2 iteration as the most effortless entry point to integrate complexity. Within sectors, more channels for communication and collaboration might exist facilitating the integration of knowledge and perspectives (Menk et al., 2022). Additionally, as there is growing attention in research to better understand hazard interactions (Zscheischler et al., 2020; Poljansek et al., 2021), the proposed framework to create (intermediate-) adaptive pathways for single- or multi-hazard-related planning can already serve as useful information for sectoral users. This is expected to support the understanding of different sectoral stakeholders for multi-risk and to increase their willingness to participate in the process (Butler et al., 2020; Zandvoort et al., 2019; Head, 2019). On the other hand, the integration of cross-sectoral knowledge might cause additional challenges owing to confidentiality requirements and lack of trust across sectors (Scolobig et al., 2017) which complicates integrating knowledge and complexity. Nevertheless, DAPP-MR and the identified guiding questions for analysis also permit to adapt the order of integrating knowledge across the three stages to a specific context (i.e. first integrating information regarding a specific hazard across sectors). One drawback of the proposed arrangement is the additional time required to revisit multiple analytical steps adapting and extending the original system and problem definition while going through the stages. Additionally, starting from a traditional single-sector, single-hazard perspective requires special consideration to avoid a common bias of too narrow a focus on traditional siloed system/problem definition, and choices of scenarios and plausible policy options (Wright et al., 2019; Shepherd et al., 2018; Stanton and Roelich, 2021).

While DAPP-MR primarily aims to guide the analysis of complex multi-risk systems, its set-up is expected to have implications on the distributive justice of the designed pathways and on the power dynamics affecting the design process. Distributive justice across present stakeholders (intragenerational equity) and with the consideration of future stakeholders (intergenerational equity) addresses the just distribution of negative and positive outcomes (Vermunt and Törnblom, 1996). Jafino et al. (2021) highlight the importance to disaggregate information regarding actors, their values, performance metrics, their policy options, and the multi-temporal dimension of all these characteristics to allow for the assessment of distributive justice. Although some of these requirements are inherent to DAPP (e.g. multi-temporal dimension of performance metrics), the staged approach explicitly requires disaggregating information per sector, and can therefore be expected to enhance the capability to account for distributive justice of decision-making. At the same time, if sector systems are not adequately differentiated into its multiple stakeholders, the aggregation on the sector level might inhibit accounting for distributive justice within sectors.

Power dynamics, on the other hand, play a much more relevant role in complex multi-risk systems than in traditional DAPP applications. In a multi-sector system, it is relevant to identify who has i) the power to take a certain policy action, ii) the power over other sectors (or stakeholders within) to initiate/inhibit certain decisions, and iii) the power to collaboration to take specific action (Avelino, 2021). In DAPP-MR, these different perspectives on power are also stagewise explored within and across sectors. This approach also allows us to investigate enabling and constraining powers within sectors and the system (Avelino, 2021) as well as power relations and corresponding power dynamics between stakeholders and sectors (Avelino and Wittmayer, 2016). At the same time, putting the sector perspective first (stage 1 and stage 2) before integrating to and identifying preferable complex multi-risk pathways might influence how stakeholders use their powers in the design and implementation process. It is plausible that complex multi-risk pathways are designed less favorable for one (or multiple) sectors to achieve better outcomes for everyone. Consequently, sectors might be incentivized to diverge from the complex multi-risk pathways for their own interests and therefore pose challenges to other sectors (Gold et al., 2022). Dealing with these circular feedbacks from a systemic perspective is thus required when developing adaptive plans, e.g. by means of agreements, compensations, or alternative compromises.

## Testing the framework in a stylized case

As introduced in the previous section, each stage concludes by identifying a set of preferred pathways accounting for different degrees of interaction and complexity. As such, DAPP-MR builds on existing expertise and provides intermediate results already relevant for sectoral DRM (Poljansek et al., 2021). In addition to the staged approach, the focus per analytical step needs to be adjusted to capture the additional information as presented in Table 1. A set of indicative questions to guide the analytical steps of the proposed DAPP-MR is provided in Table S1. We used a stylized case (see ‘STAR Methods’ section and Tables 2 and 3 for description) to give illustrative pathways maps and scorecards for each of the three stages, to investigate how the existing key elements of DAPP can deal with the increased complexity. The stylized case consists of two interconnected sectors (S1, S2) and two interacting hazards (H1, H2). Each of the sectors has two policy options in its portfolio to deal with H1 and H2. As discussed in Implications of interdependencies for the timing of adaptation tipping points and opportunity tipping points, combining the effects of interactions between multiple single-sector, single-hazard pathways may be very complex. The use of scorecards can play a central role in this process of short-listing promising sector-specific pathways throughout the staged approach.

**Table 2:** Indicative questions and answers to characterize the stylized case

Indicative questions	Answers to characterize the stylized case
How are different sectors linked to each other? What are the main types and channels of interaction under normal conditions?	S1 and S2 are mostly independent regarding the decisions they can take.
What type of multi-hazard interaction should be considered?	H1 increases probability of H2 occurring
What are the effects of multi-hazard interactions on ATPs?	S1: ATP regarding H1 remain unchanged; ATP regarding H2 changes (timing of ATP occurs earlier)
	S2: ATP regarding H1 remain unchanged & ATP regarding H2 changes (timing of ATP occur earlier)
Do opportunities arise by accounting for multi-hazard interactions?	S1: no opportunities arise
	S2: opportunity to implement a new policy option directed toward H1 (path-dependent on E as a previous policy option)
Do opportunities arise by accounting for multi-sector interactions?	Opportunity to implement a new policy option with strong synergies regarding both hazards (ATP for S1-H1 and S1-H2 won’t be reached in the planning horizon, action E benefits from new policy option)
How do we measure the success of the complex system?	Cost, Target effects regarding both sectors, cooperative stability, social acceptability, equity

**Table 3:** Trade-offs and synergies of policy options of different sectors S1 and S2 regarding the different hazards H1 and H2

			S1				S2			
			H1		H2		H1		H2	
			A	B	C	D	E	F	G	H
		A		0	0	0	0	0	0	0
	H1	B			B benefits from C	0	E benefits from B	0	0	0
		C				0	0	0	0	0
S1	H2	D					D impeded by E	0	0	0
		E						0	0	0
	H1	F							0	0
		G								0
S2	H2	H								

Zero represents no interaction between the policy options.

### Stage 1 pathways map and scorecard (single-sector, single-hazard pathways)

**Figure 5:**
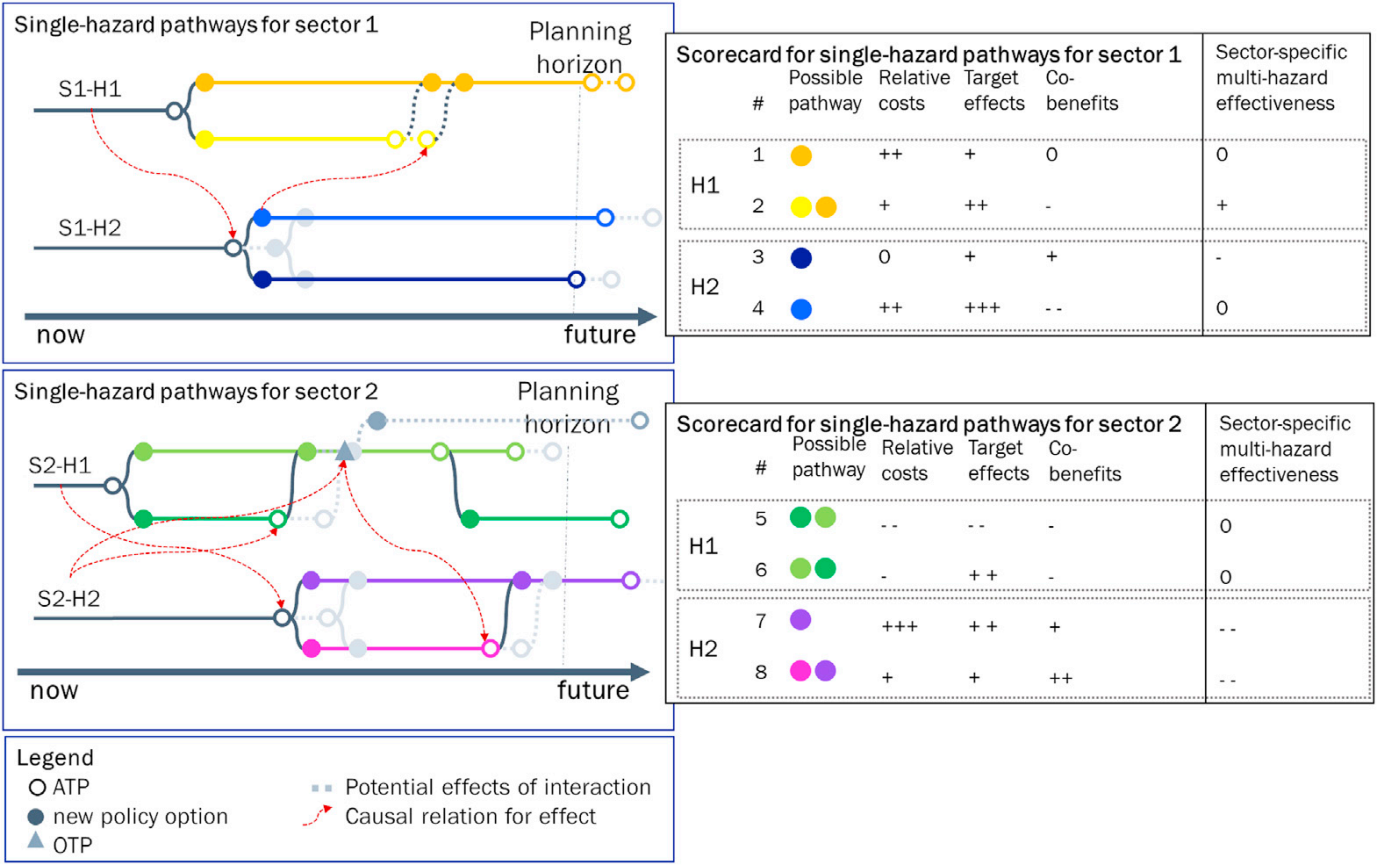
Pathways maps and scorecards for single-hazards per sector (S1-H1, S1-H2; S2-H1, S2-H2) with interactions between pathways shown in gray colors and/or dotted lines indicate effects owing todue to sector-specific interactions The vertical line is the planning horizon from the present until the system performance should be maintained. Scorecard consists of illustrative evaluation criteria. ”MH effectiveness” captures trade-offs and synergies of possible pathways regarding multiple hazards.

For the stylized case, two pathways maps (where each consists of two single-hazard, single-sector pathways) can be created (S1-H1, S1-H2; S2-H1, S2-H2) (Figure 5). These pathways maps can be used as an intermediate result to inform single-hazard risk DRM, but also to identify sector-specific interactions between the single-hazard risk management pathways. Correspondingly, the scorecard can be used to identify preferred pathways for single-hazard risk DRM using evaluation criteria as commonly used in existing DAPP applications. Furthermore, additional criteria can be considered to evaluate the multi-hazard effectiveness of and interaction across policy options to screen out inadequate policy options. As a result, the possible pathway 4 (PP4) seems promising given its good hazard-specific performance as well the potential synergy regarding policy option B (yellow). Conversely, PP5 performs inadequately as an ATP is reached before the end of the planning horizon with no additional policy option available to be implemented. The performance of PP5 can be expected to be further reduced owing to multi-hazard trade-offs (which result in earlier timing of reaching an ATP). Stage 1 maps are useful for understanding and discussing the interconnectedness between different single-sector, single-hazard pathways. However, given that comparisons can only be done one by one, it is difficult to derive conclusions about which policy options and sector-hazard-specific pathways yield higher synergies than trade-offs across sectors and hazards.

### Stage 2 pathways map and scorecard (sector-specific, multi-hazard pathways)

**Figure 6:**
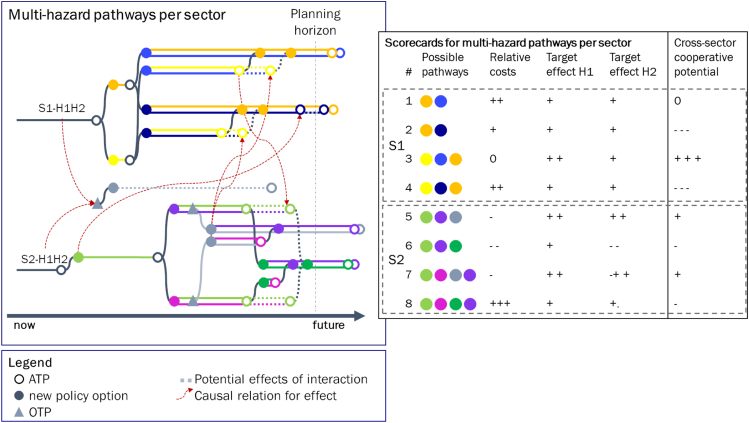
Pathways maps and scorecards for multi-hazards per sector Gray colors and/or dotted lines indicate effects owing to cross-sector, cross-hazard interactions. The vertical line is the planning horizon from the present until the system performance should be maintained. Scorecard consists of illustrative evaluation criteria.

Single-hazard pathways for each sector are integrated into two single-sector, multi-hazard pathways (S1-H1H2, S2-H1H2) by accounting for the specific interactions identified in the previous stage as visualized in Figure 6. For example, it was identified in the previous stage that a new OTP may emerge for S2 when considering a multi-hazard setting. Moreover, PP5 from Figure 5 is not considered in stage 2. The effects and performance of PPs including the opportunity policy option are evaluated as part of stage 2. These second-stage pathways integrate the perspectives and interests of each sector separately. As such, these pathways are useful to develop sector-specific pathways to manage multi-hazard risk. Alternatively, they can be used to discuss the implications of interactions between the single-sector, multi-hazard pathways. For example, PP2 performs well when just using a single-sector, multi-hazard perspective. However, in case cross-sectoral interactions are accounted for, these pathways could perform inadequately, making it a less promising pathway in stage 3.

### Stage 3 pathways map and scorecard (complex multi-risk pathways)

After characterizing the corresponding additional policy options related to the new OTPs, the single-sector, multi-hazard pathways can be integrated into multi-hazard, multi-sector pathways (S1S2-H1H2). However, fully integrated complex multi-risk pathways maps lack the required simplicity for stakeholders to support decision making (see Figure S1). The amount of information in the pathways maps can overwhelm an end-user visually and thus miss its purpose. As visible in Figure 6, interactions between partly integrated pathways (single-sector, multi-hazard) already become increasingly complex and difficult to understand. Similarly, while stage 1 and 2 pathways maps and scorecards are still explicitly relevant for the stakeholders in a specific sector, the information derived from stage 3 is mostly relevant for an overarching decision-maker (e.g. a public authority) to steer DRM in line with overarching good governance principles, including effectiveness (efficiency, and subsidiarity), equity (inter- and intragenerational), feasibility (political, technical and legal) and public acceptance (Aven and Renn, 2010; Virtudes, 2016; Florin and Bürkler, 2018; Chereni et al., 2020).

**Figure 7:**
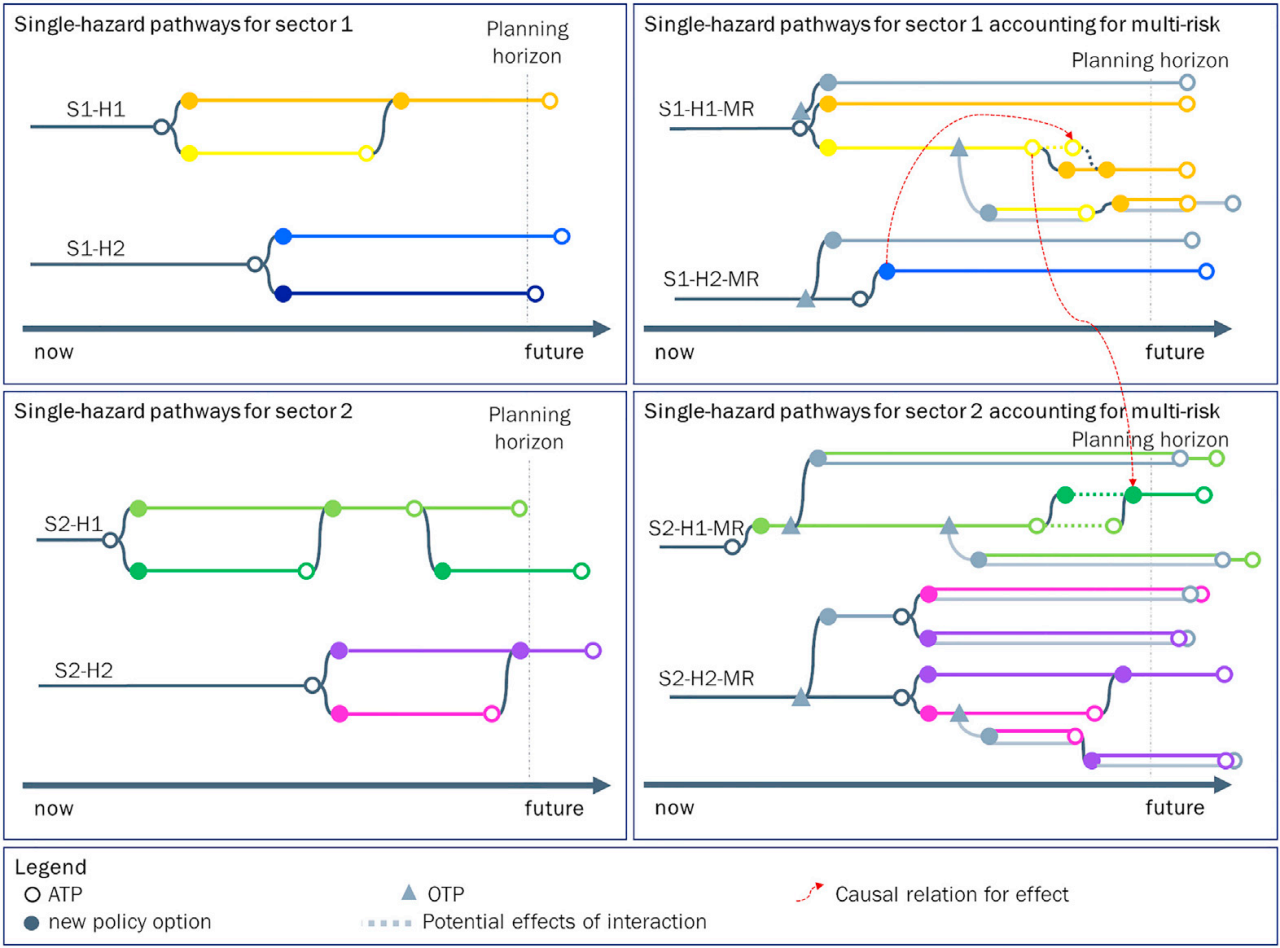
Promising single-hazard pathways per sector without (pathways maps on the left) and with accounting for multi-risk interactions (pathways maps on the right)

As such, stage 3 pathways maps (complex multi-risk) are necessary intermediate results, which are necessary for the creation of the corresponding scorecard, but not as a means to visualize potential long-term multi-risk strategies. The resulting scorecard of stage 3 in combination with the pathways maps of stage 2 can be used to disentangle the interconnections across hazards and sectors for each individual sector and hazard, as shown in the right column of Figure 7. These multi-risk-informed pathways are informative for sectoral decision-makers without the need to deal with the full complexity of combined multi-risk pathways as differences between the single-hazard pathways per sector with and without accounting for multi-risk interactions can be compared and further investigated. These differences may occur in terms of the number of available policy options and timing of their ATPs and OTPs.

## Conclusions and limitations of this study

Interactions and interdependencies amongst natural hazards and sectors exacerbate risk across sectors, space, and time. Ongoing climate change and socio-economic developments require that disaster risk management strategies apply long-term perspectives to account for trade-offs and synergies driven by these changing interactions to avoid maladaptation. Tools to support risk management and adaptation need to be adjusted to address this increasing complexity. This article proposes DAPP-MR as a tailored version of the analytical framework of DAPP to design adaptive DRM pathways in a complex and dynamic multi-risk context. DAPP was used as a promising basis for the framework development as it combined relevant elements for risk-informed, precautionary, and discursive strategies. We analyzed relevant aspects of multi-hazard, multi-sector systems to inform the development process of the new framework. We reason that a pathways framework to manage complex multi-risk systems needs to explore trade-offs and synergies of policy options across multiple interacting hazards, across contested objectives of multiple sectors, and accounting for interactions with other policy options. Furthermore, we also show that ATPs and OTPs are useful tools to capture the effects of interactions between hazards, sectors, and policy options.

The tailored framework uses the existing analytical steps of DAPP (Haasnoot et al., 2013), but extends the scope of each analytical step for multi-risk DRM addressing multiple hazards and sectors. This tailored version of DAPP is expected to address the complexity of multi-risk by guiding stakeholders through a stepwise integration of knowledge, perspectives and evaluation boundaries starting from a sector-focused entry point. As a result, developed pathways maps for different stages of integration can capture trade-offs and synergies across hazards and sectors and help designing multi-risk informed pathways. Although DAPP-MR has been tailored through the lens of long-term disaster risk management, it could also be applicable in comparable contexts characterized by multi-objective problems and highly interconnected and interdependent systems such as complex environmental systems, in the field of multi-sector dynamics (Reed et al., 2022) or for the development of climate-resilient development pathways (Werners et al., 2021a). Nevertheless, several aspects of DAPP-MR require further research and reflection for the operationalization of the framework as elaborated in the following paragraphs.

### Consideration of further relevant elements and concepts of Dynamic Adaptive Policy Pathways

The stylized case provides a first-order analysis of the utility of DAPP-MR under ideal “lab conditions” and without stakeholder involvement. Thus, it may not represent real-world situations. Although the stylized case suggests that both scorecards and pathways maps could be helpful to visualize and evaluate pathways, this conclusion should be confirmed by more detailed case studies and interactions with stakeholders. At the same time, testing of DAPP-MR did not include considerations on how to deal with contested objectives or the unwillingness of sector(s) to agree upon a multi-risk pathways strategy in case their stage 1 or stage 2 pathways maps are more satisfactory. Similarly, the process of identifying ATP’s and OTP’s has been neglected which can also have complex dependencies across the current state of the system, objectives and inter-actor conflicts/cooperation (Trindade et al., 2019; Gold et al., 2022). The resulting implications for the process of developing adaptive plans, notably signals and triggers to initiate decision points are also open questions. A starting point could be an approach proposed by Stephens et al., (2018). They link signals and triggers directly with ATPs and use the number of certain hazard events exceeding different thresholds within a specific monitoring period to initiate and take timely action.

### Identifying a toolset for the implementation of DAPP-MR

Given the required extent of information to be collected, organized, analyzed, and comprehensively presented to support good multi-risk governance (Scolobig et al., 2017), useful tools and methods to aid this process should be investigated. As we discussed that complex multi-risk systems have high degrees of interactions, interdependence, and uncertainty (Relevant aspects to characterize multi-risk systems) on one hand, and showed that already very simple systems can get rather complex in an analysis (Testing the framework in a stylized case) on the other hand, it is questionable, if DRM pathways for complex multi-risk systems can be designed in a purely qualitative, narrative-driven sense. Conversely, computational methods and tools could be necessary to account for and keep track of the different hazard-, sector- or policy-driven influences for example on the timing of reaching ATPs, or multi-temporal dimensions of system interactions. A promising starting point could be elements and tools from other approaches supporting decision-making under deep uncertainty (Kwakkel and Haasnoot, 2019). For example, model-based elements of Many-Objective Robust Decision Making (MORDM) (Kasprzyk et al., 2013) could be helpful for navigating the complexity of generating and evaluating pathways (Lawrence et al., 2019). Robust Decision Making makes use of models to simulate the implications of assumptions. In combination with approaches of scenario discovery (Groves and Lempert, 2007), it identifies relevant uncertainties (e.g., from multiple hazards) and can stress-test strategies against these scenarios to identify robust decision and contingency options. This has for example been incorporated into the Deep Uncertainty pathways framework (Trindade et al., 2019) which has been developed to discover robust pathway policies in the context of multi-actor systems.

Nevertheless, while DAPP-MR (with the right tool set) could provide support to find solutions for “difficult-to-answer” questions, complex multi-risk systems also face the challenge that they are wicked (Rittel and Webber, 1973) meaning “difficult to define problems.” In our highly interconnected and interdependent society, the problem definition (i.e., what types of compounding/cascading hazards could interact in combination with growing multi-sectoral demands, or which elements do we include as exogenous forcing or within endogenous dynamics in our system definition) already introduces significant uncertainty and ambiguity (Ringsmuth et al., 2022; Srikrishnan et al., 2022). A challenge remains whether informative pathways of actions for navigating the problem can be developed in such difficult and highly uncertain systems. Hence, while methods exist to investigate the relevance of multi-hazard interactions for risk management to avoid unnecessary complex analysis (Liu et al., 2015), additional approaches might be required to help identifying the upper limits of considerable complexity in light of uncertainty.

### Embedding DAPP-MR in practical decision-processes

Recent research shows that successful multi-risk DRM requires an inter- and transdisciplinary approach (Schweizer and Renn, 2019). This means that knowledge from various natural, technical, social, and political science disciplines (interdisciplinarity) is combined with local knowledge and practices to enhance the integrative and adaptive capabilities of risk governance processes. If adequately initiated and managed, such processes can result in the co-production of knowledge, new relationships between involved stakeholders, changes in institutionalization, and new practices or policies (Wyborn et al., 2019). Conversely, if representatives in such co-production processes are not diligently selected to represent a variety of perspectives (Klenk et al., 2017; Dilling and Lemos, 2011) and carefully managed to account for institutional characteristics (e.g. inequalities of power and resources; Sutherland et al., 2017) the outcomes can be suboptimal (Wyborn et al., 2019). These issues related to co-production processes should be accounted for in approaches to support multi-risk DRM together with the attribution of multi-hazard and multi-sector considerations. DAPP-MR implicitly assumes a functional and meaningful co-production process to be used for inter- and transdisciplinary collaboration across sectors and hazards to design pathways for complex multi-risk. Consequently, guidance on tailoring a co-production process to the application for DAPP-MR is still needed for an operational decision support tool for complex multi-risk DRM.

## STAR★Methods

### Key resources table

This paper did not use any software, models, original source data for the analysis.

### Resource availability

#### Lead contact

Further information and requests for resources should be directed to the lead contact, Julius Schlumberger (Julius.schlumberger@deltares.nl).

#### Material availability

This study did not generate new datasets.

### Method details

#### Example of a multi-sector system as referred to in Introduction

In a study area, the sectors “transport and infrastructure” and “agriculture” are defined as a multi-sector system. The “transport and infrastructure” sector is defined to consist of three sub-systems: “railways”, “motorways”, and “shipping”. Each of these sub-systems consist of multiple elements. For example, “railways” is made up of elements at risk (“railway tracks”, “train stations” and “train equipment”) and stakeholders (“public transport operator”, “freight traffic operator”). These elements at risk and stakeholders could have interrelations amongst each other (in terms of use, maintenance responsibilities etc.), across the sectoral sub-systems, cross-sectoral with regards to the elements and stakeholders of the “agriculture” sector, and beyond the sectoral and spatial boundaries of the study area.

#### Tailoring DAPP toward DAPP-MR

To further develop DAPP-MR we use a method that is inspired by findings of McMeekin et al. (2020) who investigated common practice to develop methodological frameworks, following three steps: 1) characterize DAPP as a promising basis for framework development in a multi-risk setting, 2) identify relevant aspects that should be included in a DRM pathways framework for complex and dynamic multi-risk and 3) develop and test DAPP-MR. While McMeekin et al. (2020) proposed an additional, concluding development step of evaluation for example based on a case study, it is beyond the scope of this study to evaluate DAPP-MR as the application in a real-world test case would itself comprise a full study, given the complexity of the topic. The following sections will give more context and specify methods used in the three development steps used in this research.

The process of developing the DAPP-MR framework was the key focus of this paper. Given the limited availability of methodologies guiding such developments, it is difficult to assess whether our approach performed well in comparison. We informed the development process mostly via findings from a literature review, discussions between the authors and input from other experts. The top-down approach used to identify relevant aspects in terms of conceptual, qualitative or quantitative discussions was chosen to allow for the widest application of the framework, given that various case studies would have different and unique interdependencies and dynamics that need to be considered. Similar approaches have been used by other authors (see e.g., de Angeli et al. (2022)) and are in line with the common practice of developing frameworks (McMeekin et al., 2020).

#### Characterize DAPP as a promising basis for framework development

DAPP was identified as a promising basis for framework development through discussions with experts from the disaster risk community and sectoral practice as part of the activities of the MYRIAD_EU project. MYRIAD-EU (*Multi-hazard and sYstemic framework for enhancing Risk-Informed mAnagement and Decision-making in the EU* is a EU Horizon 2020 project taking place from 2021 to 2025 which aims to catalyze a pardigm shift so that decision-makers will be able ”to develop forward-looking DRM pathways” assessing trade-offs and synergies of strategies under complex multi-risk conditions (Ward et al., 2022). As part of the agenda of the first two annual meeting of the 17 consortium partners and a workshop engaging the wider research community to present and discuss first findings of MYRIAD-EU with about 30 experts from disaster risk research and sectoral practice, challenges of integrating multi-risk considerations in risk management practices were discussed. Additional reflections on DAPP as a suitable basis for the framework were derived from eight 1-h semi-structured interviews (Rubin and Rubin, 2012) with representatives from the energy, finance, agriculture, ecosystems and transport sectors, along with other experts from the disaster risk reduction context, taking stock of the existing risk management practices and perspectives toward multi-risk governance. The interviews were prepared, conducted and evaluated in line with Hove and Anda (2005) and in accordance with the Ethics Plan of MYRIAD-EU. A set of 10 guiding questions were used to initiate the discussion:

• With which types of natural hazards are you dealing with on a regular basis in your role/organisation, if any?
• Given the definition stated earlier (UNDRR, 2017), what is your understanding of and experience with multi-hazards, multi-risks? Could you give some examples?
• In your organization and/or network, are natural hazards and risks considered individually or in interaction in the disaster risk management cycle (response, recovery, mitigation, preparedness)? For example, think about the following scenarios: an earthquake triggers multiple landslides or intense rainfall and storm surges occurring simultaneously result in extensive inland and coastal flooding.
• a) Are you aware of any policies or governance processes taking into account interactions between natural hazards? b) What specific benefits and opportunities do they bring, if any?
• Can you share examples of (local/regional/national/EU-wide) good practices that consider multi-hazard interactions and multi-risk as part of a risk management strategy? For example, think about the institutions that have responsibility for assessing, warning for, and managing different hazards, and the procedures and processes in place for managing multi-risk events.
• Are there any barriers or challenges you think decision-makers are facing in implementing multi-hazard, multi-risk management guidelines and policies in sectors/areas you are familiar with? Can you give some examples?
• What potential trade-offs or synergies do you anticipate or have experienced in the development or implementation of policies and guidelines that take into account multi-hazard, multi-risk in DRM actions?
• a) In your sectoral policies and strategy plans, do you consider dependencies or linkages between different sectors and if so, b) do these policies and plans take into account potential interactions between natural hazards? Please give some examples, if possible.
• To your knowledge, what tools, models, and frameworks are used in your sector to support multi-hazard, multi-risk assessment and management? If possible, please give some examples keeping in mind their potential inclusion in a Wiki-style platform (WP1, Task 1.2).
• Is there any further information or knowledge you would like to share with regards to policy, policy-making processes and governance for multi-hazard, multi-risk management?

Interviewees were invited to share initial written reflections before the interview. Interview responses were analyzed based on notes taken during the interviews.

#### Collecting evidence to inform the development of DAPP-MR

An integrative literature review (Snyder, 2019) was conducted to identify key aspects of multi-hazard and multi-sector systems and critically analyze their interrelations to further assess requirements to a DAPP-MR. This method has been reported to facilitate the development of new theoretical frameworks in the context of both mature and emerging topics (Snyder, 2019). We used a set of specific questions to structure the review and identify search terms and relevant literature:

• How are the terms *multi-hazard*, *multi-sector*, *multi-risk*, compounding hazards/risks (and synonyms) defined?
• What elements are frequently mentioned to characterize these terms?
• What approaches accounting for multi-hazard dynamics are currently available in the context of DRM?
• What concepts and approaches are currtly available to address multi-sector dynamics?
• What are the main challenges of multi-sector dynamics?

Relevant peer-reviewed literature in English were identified in the Google Scholar database (about 200 papers). As multi-hazards and multi-sector dynamics are emerging fields, we biased the research toward recent publications (>. 2015). By clustering the papers, we found the following themes in relevant aspects to characterize multi-risk systems: 1) Effects of multiple interacting hazards, 2) Dynamics and interdependencies of sectors, 3) Trade-offs and synergies of DRM policy options across different sectors and spatial and temporal scales. These themes are used to present the findings in Relevant aspects to characterize multi-risk systems.

#### Development and testing of DAPP-MR

We followed an iterative approach to develop DAPP-MR. We first investigated which relevant multi-risk aspects are already accounted for in the DAPP framework and which additional aspects need to be addressed. These findings were used to identify whether the existing analytical steps of DAPP are sufficient to incorporate all relevant multi-risk information for DAPP-MR. As a final step, it was analyzed if DAPP-MR can deal with the increased complexity of multi-risk settings most effectively. For this, different framework versions were tested and evaluated using a stylized case. The testing focused on the development and evaluation of the DRM pathways for complex, dynamic multi-risk. It investigated the suitability of key DAPP concepts (ATPs, OTPs, pathways maps and scorecards) to deal with the complexity of multi-risk settings. It was beyond the scope of this paper to test and evaluate the framework in a full (real-world) case study. Therefore, a stylized case was characterized based on some of the indicative questions provided in Table S1. It consists of two interconnected sectors (S1, S2) and two interacting hazards (H1, H2). Each of the sectors has two policy options in their portfolio each to deal with H1 and H2. The Figure S2 presents the single-hazard pathways per sector that will be considered for the testing. Details regarding the system characterization are summarized in Tables 2 and 3.

## Data Availability

• This paper did not use any data or code for analysis. • This paper does not report original code. • Any additional information required to reanalyze the data reported in this paper is available from the lead contact upon request
