# Supplementary material for: Proposing DAPP-MR as a disaster risk management pathways framework for complex, dynamic multi-risk
Source: iScience. 2022 Sep 30;25(10):105219. doi: 10.1016/j.isci.2022.105219 (PMC9579022; doi:10.1016/j.isci.2022.105219)
Supplement: Document S1. Figures S1 and S2 and Table S1 [file mmc1.pdf]

iScience, Volume *25*

Supplemental information

Proposing DAPP-MR as a disaster risk management pathways framework for complex, dynamic multi-risk

Julius Schlumberger, Marjolijn Haasnoot, Jeroen Aerts, and Marleen de Ruiter

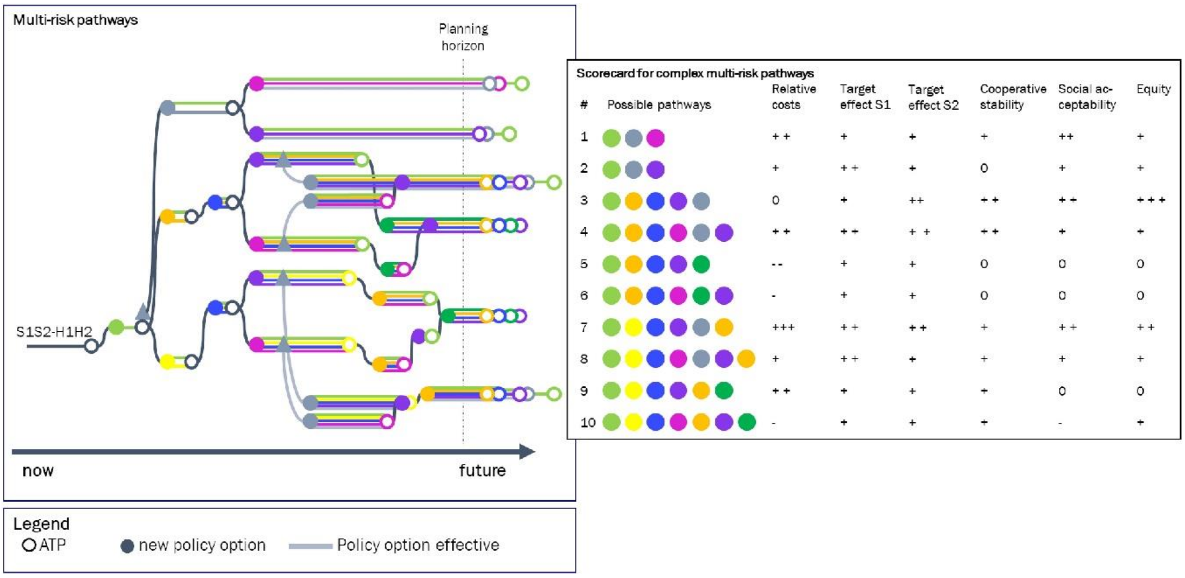

Figure S 1. [Related to in Section 5 ('Stage 3 pathways map & scorecard (complex multi-risk pathways)] Stage 3 pathways maps. Pathways maps and scorecards for multi-risk (multiple interacting hazards (H1, H2), and sectors (S1, S2). Gray colors and/or dotted lines indicate effects due to cross-sector, cross-hazard interactions. The vertical line is the planning horizon from the present until the system performance should be maintained. Scorecard consists of illustrative evaluation criteria.

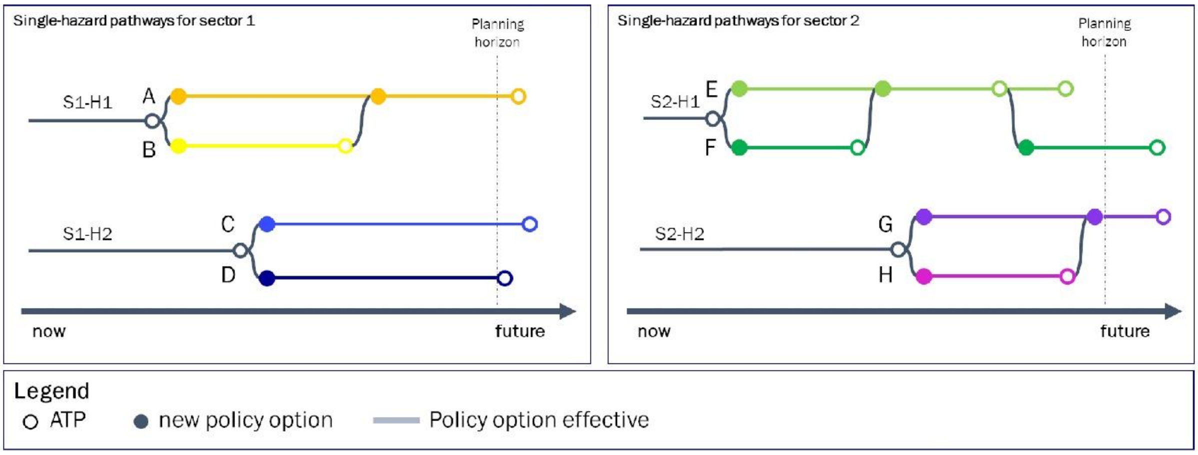

Figure S 2: [Related to STAR method section] Visualizing single-hazard, single-sector pathways of stylized case. Single-sector, single-hazard pathways (S1-H1, S1-H2, S2-H1, S2-H2) used as the basis for the development of DRM pathways for multi-risk of different combinations of policy options (options A, B, C, D, E, F, G, H).

Table S 1: [Related to Section 5 ('The DAPP-MR framework')] Detailed description of analytical steps in DAPP-MR. For the detailed description of the analytical steps in DAPP-MR, sets of indicative questions were identified that need to be answered as part of the seven steps and three stages. They are not yet tested in practice and are therefore likely to change. Since language and different terminology in different contexts might pose barriers for the universal use of the question, they should be understood as indicative guiding questions that require context-specific adjustments.

	**Sub-step**	**single-sector, single-hazard**	**single-sector, multi-hazard**	**multi-sector, multi-hazard**
Decision context	System definition	• How do you define your sector and its main functions? What elements and stakeholders are relevant?<br>• What are the spatial dimensions of the study area and the relevant planning horizon?		• How are the spatial dimensions and planning horizons of different sectors overlapping?<br>• How are different sectors linked to each other? What are the main types and channels of interaction under normal conditions?
	Participatory scoping	• How do you characterize the roles and responsibilities of these stakeholders within your sector? What are key enablers/constrains of specific roles and responsibilities?<br>• What are the overarching short-term and long-term objectives for your sector?<br>• What factors are influencing the objectives?<br>• Do you identify objectives that are conflicting with each other?<br>• What roles and responsibilities regarding risk management are present within your sector?<br>• What challenges are expected to persist or increase in future?	• How are responsibilities for the assessment, management, or communication of different considered hazards (relevant for multi-hazard) distributed within the stakeholders of your sector?	• Are there conflicts or similarities between the objectives of different sectors?<br>• How are responsibilities, resources, and power for cross-sectoral decision-making distributed?
	Hazard identification	• Which natural hazards pose significant threats to your sector today? Which natural hazards are expected to be directly impacting your sector in the future?<br>• What are drivers of these hazards?<br>• What hazard related impact drivers can be expected?	• What type of multi-hazard interactions have you observed in your sector/study area before?<br>• What type of multi-hazard interactions presented in literature do you want to take as a plausible scenario for this planning exercise?<br>• How does the interaction play out (beyond already identified system dynamics)?	• Which (multi-)hazard scenarios are relevant for multiple sectors?
	Uncertainties	• What are the key sources of uncertainty that worry your sector?<br>• Which of these uncertainties do you want to account for in your planning?<br>• What potential developments and changes of your sector (in terms of key stakeholders, functionality, key characteristics etc.) seem plausible for your sector during the planning period?<br>• What transient scenarios can be used to capture the identified key uncertainties (and hazard)	• What sources of uncertainties play a role in the characterization of hazard interactions?<br>• Which key uncertainties should be considered and how?	• What diverging understanding of the cross-sectoral dynamics (and dependencies) exists? What set of representative cases can be developed to capture the width of significantly different opinions?<br>• What potential changes of multi-sector dynamics (in terms of key stakeholders, interactions, dependence etc.) seem plausible during the planning period?<br>• What adjustments to the transient scenarios are necessary to merge those developed for single-sector perspectives?

Assess vulnerabilities and opportunities	Characterize hazard-related	• What hazard-related impact drivers are relevant for your sector?	• What combinations of hazard-related impact drivers are relevant for the considered multi-hazard scenarios?<br>• How are these multi-hazard related impact drivers develop over time?<br>• Are there spatial differences regarding the (dynamics of) multi-hazard related impact drivers?	
	Characterize exposure	• What elements at risk are relevant to be considered?<br>• What are the potential effects of impact drivers on elements-at-risk?	• How do multi-hazard interactions change intensity and extent of hazard-related impact drivers and thus affect the set of elements at risk to be considered?<br>• How do recovery dynamics influence the values of certain elements exposed to potential consecutive hazards?	
	Characterize vulnerabilities	• What factors are determining the vulnerability of elements-at-risk?<br>• What factors influence the vulnerability of stakeholders owning certain elements-at-risk?<br>• What are the consequences of impacts on stakeholders?<br>• How long do impacts (or effects of impacts) pertain in time?	• How is the (updated) set of elements at risk vulnerable to the multi-hazard related impact drivers?<br>• How do recovery dynamics influence the share of maximum impact potentially caused by consecutive hazards?	• How do cross-sectoral interdependencies influence the vulnerability of elements at risk to multi-hazard related impact drivers?<br>• What types of cross-sectoral interdependencies cause additional impacts to specific elements at risk?<br>• What impact spill-over chains can be identified? What are impact thresholds and temporal and spatial dynamics of these impact spill-over chains?
	Identify ATPs	• What conditions would lead to irreducible system change (adaptation tipping points)?<br>• What conditions would trigger adaptive actions being implemented?<br>• What additional indicators are relevant to specify conditions in your sector that fail to meet its targets (caused by natural hazards but also other drivers)?<br>• Remain the ATPs constant over time (depending on a specific transient scenario)?<br>• Assuming that no DRM measures would be taken, would these ATPs be exceeded in short-, medium- or long-term of the planning horizon for the different single-sector, single-hazard transient scenarios?	• Do you expect that the awareness of multiple (interrelated) hazards changes the ATP thresholds identified for single-hazard considerations?<br>• Assuming that no DRM measures are taken, what would be the effect of exacerbated changes on the relative timing of the ATPs for the single-sector, multi-hazard transient scenarios?	• How do considerations of cross-sectoral influences on the vulnerability as well as the potential risk of impact-spill-over chains influence the ATP thresholds for the elements at risk?<br>• Are additional ATPs necessary to capture the multi-sector effects on vulnerability?<br>• Can ATPs for different sector (for the same (multi-)hazards) be streamlined?<br>• Assuming that no DRM measures would be taken, would these (new) ATPs be exceeded in short-, medium- or long-term of the planning horizon for the multi-sector, multi-hazard transient scenarios?
	Charact erize	• What hazard-driven conditions could leverage new opportunities for (transformational) changes in the system?<br>• What indicators and threshold values can be identified to represent these OTPs?	• Do you expect that the awareness of multiple (interrelated) hazards introduces new opportunities for (transformational) changes in the system?	• Do you expect that alignment of objectives, similarities in vulnerabilities, cross-sectoral interdependencies, or similarities regarding the timing of ATPs across sectors leads to opportunities for cooperation and coordination?

		• Assuming that no DRM measures would be taken, when would these conditions occur (in short-, medium-, or long-term)?	• What indicators and threshold values can represent these additional OTPs?<br>• Assuming that no DRM measures would be taken, when would these (updated) OTPs occur (in short-, medium-, or long-term)?	• What could be the implications of above cooperation and coordination?<br>• What indicators and threshold values could be specified to describe the conditions under which cross-sectoral opportunities could be seized?<br>• Assuming that no DRM measures would be taken, when would these conditions occur (in short-, medium-, or long-term)?
	Uncertainti es	• What sources of uncertainties play a role in the characterization of vulnerability, exposure impact effects and conditions triggering additional actions?<br>• Which key uncertainties should be considered and how?		
Identify & evaluate policy options	Developing set of preferred policy options	• What policy options are potentially useful to address the specific hazard-related impact drivers?<br>• What policy options fall in the mandate of the stakeholders of the sector?<br>• How can the policy options be characterized in terms of potential effectiveness, readiness, lead time until full effectiveness, duration of benefits, societal acceptability, governability, potential co-benefits, and potential negative collateral effects?<br>• Do the organizational implications of a certain policy option (readiness, lead time until full effectiveness…) match the required timeline for implementation according to the timing of ATPs and OTPs?<br>• Under which conditions are certain policy options (not) practical?<br>• How are policy options complementary to each other in with regards to resources (e.g. space) and effects (e.g. complementary, conflicting...)?<br>• What set of preferred policy options can be identified able to address the vulnerabilities and opportunities within the sector regarding single-hazard risk?	• What are the trade-offs/synergies of policy options across different hazards?<br>• How do vulnerabilities of policy options to different hazard-related impacts influence the effectiveness of the policy options?<br>• Can additional policy options be identified that address multi-hazard related impact drivers?<br>• How are the updated set of policy-options complementary to each other in with regards to resources (e.g. space) and effects (e.g. complementary, conflicting...)?<br>• What set of preferred policy options can be identified able to address the vulnerabilities and opportunities within the sector regarding multi-hazard risk?	• What are the trade-offs/synergies of policy options across different sectors?<br>• How do different sectors evaluate the policy options of other sectors in terms of cooperative opportunities, conflicts (e.g. in terms of spatial requirements) or trade-offs?<br>• What set of preferred policy options can be identified able to address the vulnerabilities and opportunities with regards to multi-risk?
	Uncertainties	• What sources of uncertainties play a role in the characterization of policy options?<br>• Which key uncertainties should be considered and how?		

<table>
<tr>
<td rowspan="2">Develop & evaluate pathways</td>
<td>Identifying preferred pathways</td>
<td>What sequences of policy options can be developed that perform well (e.g. make use of OTPs, avoid that ATPs are exceeded) for a wide range of transient scenarios?
Which of the well-performing pathways are preferred because of good benefit-cost ratios, target effects and co-benefits?
Which of these good evaluated pathways are also promising with regards to potential synergies for multi-hazard considerations?</td>
<td>What interaction effects (because of interactions between hazard and/or policy options) between different single-sector, single-hazard pathways need to be accounted for?
What set of preferred single-sector, multi-hazard pathways can be built from the promising single-sector, single-hazard pathways?
Which of these preferred pathways are also promising because of potential for cross-sectoral collaboration or absence of major trade-offs?</td>
<td>What interaction effects (because of interactions between sectors and/or policy options) between different single-sector, multi-hazard pathways need to be accounted for?
What set of preferred multi-sector, multi-hazard pathways can be built from the promising single-sector, multi-hazard pathways?
How do these pathways perform in terms of overarching governance principles (e.g. equity, societal acceptability...) and multitude of objectives of multiple sectors?</td>
</tr>
<tr>
<td>Uncertainties</td>
<td colspan="3">What sources of uncertainties play a role in the development and evaluation of potential pathways?
How can these uncertainties be accounted for?</td>
</tr>
<tr>
<td colspan="2">Develop Adaptive Plan</td>
<td colspan="3">What initial policy options are preferred as initial actions in a wide set of pathways?
What contingency actions should be taken to keep long-term options open depending on the evolvement of the future?
What additional investigations are required to reduce uncertainties identified during the analysis process?
Which developments and system parameters need to be monitored to inform the dynamic adjustment and stepwise implementation of the (updated) plan?
Which stakeholders are relevant for the initiation of the implementation, for the monitoring or communication? Are structures necessary to ensure the exchange of information?</td>
</tr>
<tr>
<td colspan="5">Implement strategy and Monitor strategy</td>
</tr>
</table>
